# The influence of sex on the treatment of postural tachycardia syndrome in children

**DOI:** 10.1097/MD.0000000000033951

**Published:** 2023-07-14

**Authors:** Yali Peng, Shuo Wang, Runmei Zou, Hong Cai, Juan Zhang, Yuwen Wang, Cheng Wang

**Affiliations:** a Department of Pediatric, Xiangya Hospital, Central South University, Changsha, China; b Section of Science and Education, The First People’s Hospital of Changde City, Changde, China; c Department of Pediatric Cardiovasology, Children’s Medical Center, The Second Xiangya Hospital, Central South University, Changsha, China.

**Keywords:** children, postural tachycardia syndrome, prognosis, sex, treatment

## Abstract

There are differences in postural tachycardia syndrome (POTS) incidence and manifestations in children between the sexes. However, there is limited evidence on how the gender affects the prognosis of POTS in children. This study is aimed at exploring the differences between the sexes regarding the prognosis of children with POTS. A retrospective study was conducted on children (n = 53; aged 6–14 years) who were diagnosed with POTS. All the POTS patients were given health education and autonomic function training, their water and salt intake was increased (oral rehydration salt III, 250 mL, Bid), and they were administered oral metoprolol (1 mg/kg per day) for 3 months. The prognosis was defined by the head-up tilt test results after treatment. It was observed that male and female children exhibited different trends in POTS prognosis. Further, the sex showed a stable independent effect on prognostic in children with POTS. To elaborate, females had a 503% increased risk of poor prognosis compared to males. We hence hypothesize that there is an association between the sex and the POTS prognosis in children. Female patients have a significantly higher risk of poor prognosis compared to males. A slight increase in the dose of oral rehydration salt could help lower the risk of poor prognosis in children with POTS. A higher absorption of total metoprolol, lower local concentrations, and slower metabolic excretion are documented in research in female POTS patients during treatment. It is recommended that the optimal dose of metoprolol should be lowered in female children undergoing treatment, to limit the risk of poor prognosis.

## 1. Introduction

Syncope is a clinical condition that manifests as a transient loss of consciousness caused by hypoperfusion of the whole brain and makes 1 fall to the ground due to decreased muscle tone. However, one can recover on their own in a short time.^[1,2]^ At least 1 episode of syncope has been reported in 17.37% of children aged 2 to 18 years old, with females being more likely to encounter syncope than males.^[3]^ Neurally-mediated syncope is mainly caused by autonomic nerve-mediated reflex dysregulation or autonomic dysfunction and accounts for 70% to 80% of the total cases of syncope. Postural tachycardia syndrome (POTS) is one of the common types of neurally-mediated syncope.^[4–6]^ It is characterized by an excessive increase in the heart rate (HR) after standing and chronic day-to-day orthostatic intolerance symptoms, such as dizziness, headache, chest discomfort, palpitations, blurred vision, profuse perspiration, and even syncope.^[1]^ A standing test or head-up tilt test (HUTT) can provide important evidence for the diagnosis of POTS. The possible potential pathophysiological mechanisms include partial autonomic neuropathy, hypovolemia, and the hyperadrenergic state. Further, patients often exhibit overlapping characteristics of one or more of these pathophysiological mechanisms. The approach in treating POTS is mainly based on treating the underlying pathophysiologic mechanism.^[7]^

Non-pharmacological treatment is the main intervention method for POTS, which includes health education, such as avoidance of triggers, using stockings, employing abdominal binders, taking vasoconstrictors, performing appropriate physical exercises, autonomic nervous function exercises, and increasing the intake of salt and water (e.g., oral rehydration salts [ORS]).^[1,8,9]^

While non-pharmacological treatment has significantly improved the prognosis of POTS in children, additional pharmacotherapy is necessary when the outcome of non-pharmacological treatment is not satisfactory. For example, Boris et al^[10]^ suggest that non-pharmacological treatment alone is not sufficient and that additional medication may be required to improve symptoms. The medication administered is usually ORS and metoprolol. ORS, which increases the patient’s blood volume by supplementing with water and electrolytes, has become the basic treatment approach for POTS in children^[1,11]^ and may reduce the symptoms of syncope induced by the Bezold-Jarisch reflex. Additionally, metoprolol is listed in “2018 Chinese Pediatric Cardiology Society guidelines for the diagnosis and treatment of syncope in children and adolescents” as a drug that suppresses sympathetic excitability in children with POTS.^[1]^ Although the combination of non-pharmacological and pharmacological treatments of POTS in children has been clinically effective in the majority of cases, differences in prognosis have been observed among the sexes in children with POTS. This is despite being treated with the same treatment protocol with the number of females with a poor prognosis outnumbering that of males.^[12,13]^ We hypothesize that various physical characteristics associated with sex may have an impact on prognosis, but this has yet to be documented in recent research. In this research paper, we propose to investigate the impact of sex on the outcome of POTS in children undergoing the same treatment protocol.

## 2. Methods

### 2.1. Research design

This is retrospective research to explore if the difference in sex has an impact on the prognosis of POTS in children who had the same treatment protocol. The dependent variable was the prognosis of the children treated with non-pharmacological treatment combined with pharmacological treatment. We divided them into the good prognosis group and poor prognosis group based on whether the HUTT met the diagnostic criteria for POTS after therapy and if the clinical symptoms improved.^[14]^ After 3 months of treatment follow-up, if syncope or pre-syncope symptoms^[1]^ improved and the HUTT results no longer met the diagnostic criteria for POTS it was considered a good prognosis. If the syncope or pre-syncope symptoms did not improve and the HUTT results still met the diagnostic criteria for POTS it was grouped as a poor prognosis.^[14]^

### 2.2. Research population dataset

The data of participants with newly-diagnosed POTS were non-selectively and consecutively collected from the Department of Pediatric Cardiovasology, The Second Xiangya Hospital, Central South University. Our data did not include identifiable participants’ data for protecting their privacy. Data were then compiled from Hospital’s Electronic Medical Record System. Informed consent from the participants was not required in this research protocol because its nature was that of retrospective research. The Ethics Committee of The Second Xiangya Hospital, Central South University has approved this research.

**Figure 1.**
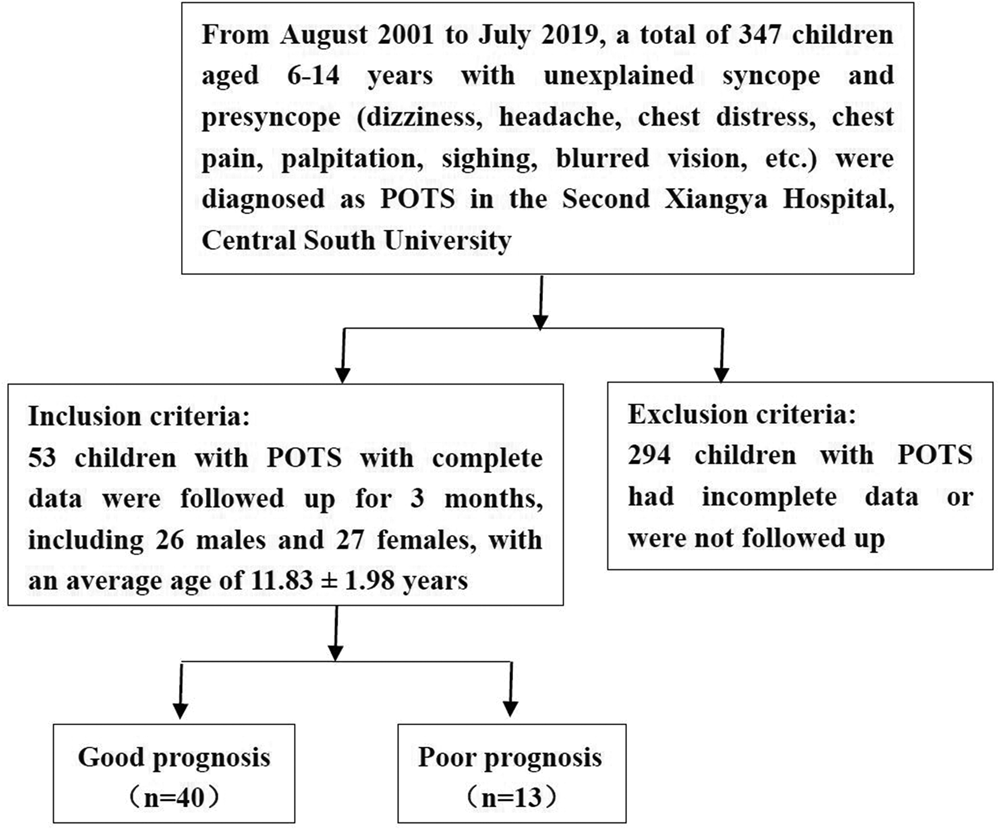
Inclusion and exclusion criteria. POTS = postural tachycardia syndrome.

A total of 347 participants were screened. The dates of inclusion and completion of participant data entry were between August 2012 and July 2019, respectively. The clinical diagnosis and treatment process of each participant were completely following “2018 Chinese Pediatric Cardiology Society guidelines for the diagnosis and treatment of syncope in children and adolescents.”^[1]^ The inclusion and exclusion criteria are showed in Figure 1. Ultimately, our dataset included 53 patients with POTS aged 6 to 14 years who completed 3 months of treatment and were followed up with HUTT. This included 26 males and 27 females (average age = 11.83 ± 1.98 years).

### 2.3. Head-up tilt test details

The diagnosis of POTS in this research involved only the baseline head-up tilt test. Research subjects discontinued cardiovascular active drugs that might affect autonomic functions for more than 5 half-lives and related foods such as coffee before the trial. Before the experiment, 4 hours of fasting was followed and drinking was prohibited. The examination time was arranged from 8:00 am to 11:00 am, the environment was quiet and the room temperature was 20°C–24°C. For the HUTT, the tilting device was the Head-up Tilt Test Monitoring System (SHUT-100) from Jiangsu Standard Medical Technology Co., Ltd. The subjects were kept supine on the tilt bed for 10 minutes, and their HR, blood pressure (BP), and electrocardiogram (ECG) were monitored and recorded. After recording the baseline ECG and BP of the subject in the prone position, the subject was tilted 60° with the head high and feet low position within 15 seconds. The HR, BP, and ECG indicators were monitored dynamically during the tilt for 10 minutes or at the time point of a positive response.^[1]^

### 2.4. Diagnostic criteria for POTS

The following were the criteria: children who were below 18 years old; orthostatic intolerance symptoms such as dizziness, headache, weakness, blurred vision, chest tightness, palpitations, hand tremor, limited movement in an orthostatic position, and even syncope; and HUTT: supine HR was normal, and, during the initial 10 minutes of HUTT, HR increased ≥40 bpm or is ≥130 bpm (in children 6–12 years old) or ≥125 bpm (in adolescents 12–18 years old), without orthostatic hypotension (BP decrease > 20/10 mm Hg).^[1]^

### 2.5. Variables

The variables used in this study can be divided into the following categories: continuous type variables: age, height, weight, metoprolol dose (mg/d), and the ORS dose (mL/d) and categorical variables: sex and prognosis.

### 2.6. Treatment protocol

#### 2.6.1. Medications included.

ORS III (250 mL, Bid) and metoprolol (1 mg/kg per day). The follow-up was after 3 months of treatment.^[15,16]^

#### 2.6.2. ORS III.

The dosage was 5.125 g/dose, Bid, was dissolved in water and given orally in divided doses.^[17]^ ORS III was manufactured by Xi’an Anjian Pharmaceutical Co Ltd (Approval no: H20090205, Specification 5.125 g per sachet). The formulation details are given here, glucose anhydrous 3.375 g, sodium chloride 0.65 g, potassium chloride 0.375 g, and sodium citrate 0.725 g. Sodium concentration 75 mmol/L, osmolality 245 mOsm/L.

Regarding metoprolol, the manufacturer was AstraZeneca Pharmaceuticals Ltd. (Approval No: H32025391, Specification 25 mg per tablet).

### 2.7. Statistical analysis

Based on whether the continuous variables were normally distributed, we presented continuous variables in this study as mean ± standard deviation (normal distribution) or quartile (P25, P75) (skewed distribution). Categorical variables were expressed in frequency or as a percentage. We employed the χ^2^ test (categorical variables), Student *t* test (normal distribution), or the Mann–Whitney *U* test (skewed distribution) to analyze the differences among different prognosis groups of POTS (clinical cut point). Univariate analysis was used to roughly evaluate the relationship between each variable and the prognosis of POTS. Further, multivariate Logistic regression was used to analyze the possible association between the sexes and the prognosis of POTS under the same treatment protocol. We constructed 3 models to illustrate the stability of this relationship. The details are given herewith. Model Iwas adjusted for none. Next, Model II adjusted for age, height, and weight. Finally, Model III was adjusted for age, height, weight, ORS dose, and metoprolol dose. To address the nonlinearity of metoprolol dose and prognosis of POTS for different sexes, a generalized additive model and smooth curve fitting (penalized spline method) were conducted. All the analyses were performed employing the statistical software packages R (version3.4.3) (http://www.R-project.org, The R Foundation) and EmpowerStats (http://www.empowerstats.com, X &Y Solutions, Inc, Boston, MA). *P* values less than .05 (2-sided) were considered statistically significant.

## 3. Results

### 3.1. Basic characteristics of participants

The basic characteristics of the study participants are presented in Table 1. The average age of the 53 selected participants was 11.83 ± 1.98 years, and 49.05% of them were male. No differences were seen between the sexes for height, weight, ORS dose, and metoprolol dose (*P* > .05). While the age was greater in females (*P* < .05), the prognosis was better in males than females (*P* < .05).

**Table 1:** Baseline characteristics of participants [n = 53, mean ± SD, n (%)].

	Male (N = 26)	Female (N = 27)	*P* value
Age (yr)	11.19 ± 1.94	12.48 ± 1.70	.013
Height (cm)	152.19 ± 14.01	154.37 ± 12.29	.550
Weight (kg)	40.98 ± 9.47	42.28 ± 10.16	.633
ORS (mL/d)	12.95 ± 3.57	12.57 ± 3.25	.633
Metoprolol (mg/d)	40.98 ± 9.47	42.28 ± 10.16	.685
Prognosis			
Good	23 (88.46%)	17 (62.96%)	.031
Poor	3 (11.54%)	10 (37.04%)	

ORS = oral rehydration salts.

### 3.2. Univariate analysis

An association emerged between sex and poor prognosis. Females had a 351% increased risk of poor prognosis compared to males (odds ratio [OR] = 4.51, *P* = .04). There was a trend towards an increased risk of poor prognosis with increasing age, height, weight, and metoprolol dose (OR = 1.24, 1.04, 1.04, 1.04). Further, a trend emerged towards a decreased risk of poor prognosis with increasing ORS dose (OR = 0.88) (Table 2).

**Table 2:** Univariate analysis for prognosis [n = 53, mean ± SD, n (%)].

Exposure	Statistics	OR (95% CI)	*P* value
Sex			
Male	26 (49.06)	1.0	–
Female	27 (50.94)	4.51 (1.07, 18.93)	.040
Age (yr)	11.85 ± 1.92	1.24 (0.86, 1.80)	.247
Height (cm)	153.30 ± 13.08	1.04 (0.98, 1.10)	.173
Weight (kg)	41.64 ± 9.75	1.04 (0.97, 1.11)	.285
ORS (mL/d)	12.75 ± 3.38	0.88 (0.71, 1.09)	.247
Metoprolol (mg/d)	41.64 ± 9.75	1.04 (0.97, 1.11)	.285

CI = confidence interval, ORS = oral rehydration salts.

### 3.3. Results of unadjusted and adjusted logistic regression

Our analyses revealed that female children with POTS had a much higher risk of poor prognosis compared to males in all 3 models. Further, this impact was stable across all 3 models, with a statistically significant difference. Overall, female children with POTS had a 503% increased risk of poor prognosis compared to males. This was an independent effect after adjusting for age, height, weight, metoprolol dose, and oral rehydration salt dose (Table 3).

**Table 3:** The relationship between sex and prognosis in different models.

		OR (95% CI)	*P* value
Model I	Male	1.0	
	Female	4.51 (1.07, 18.93)	.040
Model II	Male	1.0	
	Female	5.12 (1.07, 24.49)	.041
Model III	Male	1.0	
	Female	6.03 (1.20, 30.23)	.029

Data in the table: CI = confidence interval, OR = odds ratio.

Results variable: prognosis.

Exposed variable: sex.

Model I adjust for: none.

Model II adjust for: age, height, and weight.

Model III adjust for: age, height, weight, oral rehydration salts dose, and metoprolol dose.

### 3.4. Smooth curve fitting of the metoprolol dose and poor prognosis in children with POTS by sex

**Figure 2.**
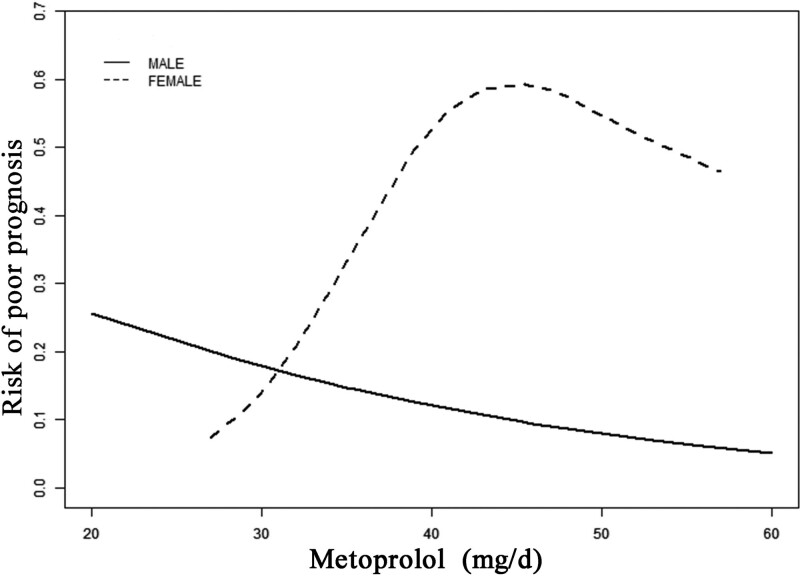
Smooth curve fitting of metoprolol dose and poor prognosis in children with POTS by sex. POTS = postural tachycardia syndrome.

In females with POTS, the risk of poor prognosis rose significantly when the metoprolol dose was increased. However, in males with POTS, the risk of poor prognosis was slightly reduced when the metoprolol dose was increased, although the difference in trend was not significant when compared to females (Fig. 2).

## 4. Discussion

Given that the pathophysiology of POTS in children is still unclear and may be affected by a combination of variables, an individualized treatment plan should be developed based on several pathophysiological processes and biological predictors.^[18]^ The prognostic outcomes for children with POTS under ORS treatment are different by sex.^[12,13]^ Although the currently reported biological markers have been documented to be clinically effective in guiding the precise treatment of POTS in children,^[19]^ the relationship between the sex and the prognosis of POTS in children has not yet been reported in the literature.^[1,20]^

In our analysis of the dataset, we found that males had a better prognosis than females’ children with POTS. As a corollary, females had a significantly increased risk of poor prognosis compared to males. After adjusting for confounding factors, these differences between the sexes still showed a strong independent effect on prognosis. In literature, Boris et al^[21]^ reported that 3 female-to-male transsexual POTS patients showed improved clinical symptoms of POTS after testosterone therapy. This may explain the better prognosis of POTS in males than in females.

In addition, the better prognosis for males compared to females may also be related to hormonal changes in the body. For instance, Stickford et al^[22]^ reported that while in females with POTS the sympathetic nerve activity is not affected during menstruation, modulated BP and vasoconstriction during tilt are responsible for orthostatic intolerance during the menstrual cycle in females with POTS. Additionally, Fu et al^[23]^ found that the menstrual cycle in POTS females could affect the renin-angiotensin-aldosterone system during prolonged standing, modulating hemodynamics during standing in POTS. Further, the mid-luteal (MLP) hyperestrogens and progesterone were associated with increased adrenaline and possibly more fluid retention, thereby improving the orthostatic tolerance in these patients. Further, Coupal et al^[24]^ also found that hormonal changes in the body during adolescence could affect orthostatic intolerance. The lower orthostatic tolerance in females compared to males is associated with increased susceptibility to autonomic dysfunction. Increased leptin, insulin, thyroxine, and increased sympathetic activity or catecholamine levels during puberty can exacerbate the abnormal increase in heart rate in patients with POTS.

We scrutinized the relationship between the ORS dose and poor outcomes in a univariate analysis and found that the risk of poor outcomes reduced as the ORS dose increased. POTS is caused by cerebral hypoperfusion in the brain due to a relative lack of blood volume, thus inducing orthostatic intolerance symptoms. ORS is a liquid suitable for gastrointestinal absorption and can rapidly rehydrate blood volume and relieve orthostatic intolerance produced by cerebral hypoperfusion in the brain.^[12]^ Therefore, a moderate increase in the dose of ORS can help to reduce the risk of poor prognosis in POTS.

We observed different responses of the sexes to different doses of metoprolol in smooth curve fitting. It was revealed that a high dose of metoprolol was harmful to the prognosis of females with POTS. Sex differences in prognosis are mediated by multiple factors. These include differences in body composition, weight, drug absorption and distribution, metabolic enzymes, and excretion pathways between males and females.^[25–27]^ The rate of absorption of metoprolol is also affected by many parameters including intestinal transit time, drug hydrolysis and lipid solubility, the pH at the absorption site, the degree of ionization and molecular weight of the medication, and intestinal motility. It was documented that the baseline and maximum amounts of gastric acid and the acidity of gastric acid were higher in males than in females during drug absorption (pH = 1.92 vs. pH = 2.59) while the pH of metoprolol was 6.0-7.0. Further, the digestive tracts of males have a lower pH than that of females, resulting in lower metoprolol absorption. Additionally, the hepatic clearance of the drug is influenced by hepatic blood flow and enzyme activity.^[27]^ Metoprolol is predominantly metabolized by the liver, and higher levels of estrogen and progesterone in females change hepatic enzyme activity. This increases metoprolol accumulation or lowers metoprolol clearance overall resulting in higher total blood metoprolol concentrations in females than in males. However, the smooth curve fitting between different sexes show that higher metoprolol concentrations significantly increased the risk of poor prognosis in female. Further, several bodily components also have an impact on drug distribution such as plasma volume, body mass index, mean organ blood flow, total body water, plasma proteins, body fat, and cardiac output. Metoprolol is a class of ionotropic medication and is fat-soluble. As female patients have more body fat than males, given the same quantity of fat-soluble drug exposure, the higher fat content in females would increase the volume of distribution and lower the drug concentration. Additionally, the high overall concentrations and low target concentrations increase the risk of poor prognosis in females. The kidney is the primary organ for the elimination of drug metabolites, and the physiological functions of glomerular filtration, tubular secretion, and tubular reabsorption vary by sex. In males, the renal clearance is generally higher than in females and metoprolol metabolites are excreted at a slower rate in females than in males. Additionally, negative feedback reduces metoprolol metabolism in the liver to some extent and thus prolongs the duration of the high-concentration levels of metoprolol in the body.

Regarding the metoprolol dosage, following “2018 Chinese Pediatric Cardiology Society guideline for diagnosis and treatment of syncope in children and adolescents” is recommended.^[1]^ Metoprolol is effective for pediatric POTS at an initial dose of 0.5 mg/(kg·d) divided into 2 administrations. The total dose of the drug should not exceed 2 mg/(kg·d). For instance, Zhang et al^[28,29]^ reported the efficacy of metoprolol [1 mg/(kg·d)] combined with ORS in the treatment of pediatric POTS and found that the efficiency at 3 months of treatment was higher in the treatment group than in the control group (80% vs 40%, *P* < .05). Further, the degree of increase in the HR of the HUTT process was significantly lower in the treatment group at the post-treatment review compared to the pretreatment baseline values. In another report, Raj et al^[30]^ found in a randomized controlled crossover trial (n = 72) that while oral propranolol (20 mg) significantly reduced tachycardia symptoms in patients with POTS, oral propranolol (80 mg) did not further improve the outcome and even worsened the condition. Oral metoprolol [1 mg/(kg·d)] may improve symptoms in POTS children who do not respond well to health education and rehabilitation exercises and whose POTS symptoms remain significant. Additionally, Arnold et al^[31]^ reported the effect of low-dose propranolol on the maximal exercise capacity in POTS patients (n = 11) and found that low-dose propranolol (20 mg) improved maximal oxygen uptake in POTS patients. However, higher doses of propranolol (80 mg) and metoprolol (100 mg) did not the improve maximal oxygen uptake. It is believed that low-dose propranolol is beneficial in improving HR modulation and organismal exercise capacity in POTS patients. This research treatment for pediatric POTS used a moderate dose of metoprolol [1 mg/(kg·d)], which both reduced the symptoms of excessive increase in HR caused by POTS and improved the maximum oxygen uptake in POTS.

To summarize, a slight increase in the amount of ORS could help lower the risk of poor prognosis in children with POTS. Given the higher absorption of total metoprolol, lower local concentrations, and slower metabolic excretion in female children with POTS during treatment, the dose of metoprolol should be lowered in female children on treatment, if appropriate, to limit the risk of poor prognosis.

## 5. Conclusions

Female children with POTS had a worse prognosis than males for the same treatment protocol. Further, the dose of metoprolol should be adjusted to account for sex variations as required.

## 6. Advantages and limitations

This research analysis investigated the possible influencing factors in the prognosis of children with POTS from the perspective of sex differences. It also analyzed the absorption, distribution, and metabolism of the drug in the body. It may offer a new way for better treatment in some female children with POTS who have a bad prognosis and also serve as recommendations for modifying sex-specific metoprolol dose protocols in children with POTS.

Regarding the limitations, the modest sample size was only aimed at studying the trends in the treatment efficacy in children with POTS according to the sex. The extent of metoprolol dose reduction for female children with POTS who do not respond well to treatment is to be further validated with an expanded sample size.

## Acknowledgements

The authors thank all the staff members in our institution.

## Author contributions

**Conceptualization:** Yali Peng, Hong Cai, Yuwen Wang, Cheng Wang, Shuo Wang.

**Data curation:** Yali Peng, Hong Cai, Juan Zhang, Yuwen Wang, Cheng Wang, Shuo Wang.

**Formal analysis:** Yali Peng, Runmei Zou, Juan Zhang, Cheng Wang, Shuo Wang.

**Investigation:** Yali Peng, Runmei Zou, Juan Zhang, Yuwen Wang, Cheng Wang, Shuo Wang.

**Methodology:** Yali Peng, Runmei Zou, Hong Cai, Cheng Wang, Shuo Wang.

**Project administration:** Runmei Zou, Shuo Wang.

**Resources:** Yali Peng.

**Software:** Yali Peng, Runmei Zou, Hong Cai, Yuwen Wang, Cheng Wang, Shuo Wang.

**Supervision:** Yali Peng, Shuo Wang.

**Validation:** Yali Peng, Shuo Wang.

**Visualization:** Yali Peng.

**Writing – original draft:** Yali Peng, Runmei Zou, Cheng Wang, Shuo Wang.

**Writing – review & editing:** Yali Peng, Cheng Wang, Shuo Wang.
